# Asymmetric effects of remittances on output in expansions and recessions

**DOI:** 10.1016/j.heliyon.2024.e39690

**Published:** 2024-10-22

**Authors:** Nikeel Nishkar Kumar, Rajesh Mohnot

**Affiliations:** a School of Economics, Finance, and Marketing, College of Business and Law, RMIT University, Melbourne, Australia; b Department of Finance, College of Business Administration, Ajman University, United Arab Emirates

**Keywords:** Remittance multipliers, Asymmetric effects, Business cycles, Expansions, Contractions

## Abstract

Using non-linear methods, we argue that current research on estimates of remittance multipliers in expansions and recessions may yield biased results by ignoring whether remittances are increasing or decreasing. We employ an extended non-linear ARDL model to decompose remittance data into positive and negative shocks during expansions and recessions with data from the top eight remittance-receiving developing countries. We find that a decline in remittances will reduce output in expansions. Second, an increase in remittances will increase output during recessions. Positive remittance shocks are not associated with output in expansions, nor generally are negative remittance shocks in recessions. The managerial implications are twofold. First, policymakers can use remittances to counteract recessions in the receiving country by implementing favorable remittance policies, such as reducing transfer fees. Second, if remittances are maintained in expansions, future recessions can potentially be managed.

## 1 Introduction

Remittances represent an important financial flow for low and middle-income countries. It has grown tremendously from US$3.3 billion in 1975 to US$653 billion in 2020 and remained resilient during the COVID-19 downturn [1]. Remittances are an important lifeblood for many households in developing countries. However, its effect on output is ambiguous [2,3] with research indicating that remittances either increase, decrease, or have no effect on output. Moreover, it is also unclear at the outset what the effect of remittances on output in economic expansions and contractions is [2,3]. Research indicates that remittances, much like government expenditures, are counter-cyclical capital flows that can act as an economic stabilizer in economic fluctuations [2,3]. However, the cyclicality of remittances can be alternated between periods of expansions and recessions in the receiving country [4,5]. Ignoring whether remittances are increasing or decreasing may yield biased results of the multiplier effects of remittances [6]. Therefore, the objective of the current study is to determine whether remittance multipliers are asymmetric during business cycle expansions and recessions in the receiving country.

To empirically determine the asymmetric cyclical effects of remittances on economic activity, we model the effects of remittances on output in expansions and recessions considering whether remittances are increasing or decreasing. Some insights are offered by the New Economics of Labor Migration (NELM) framework [7]. From NELM, the insurance hypothesis suggests that remittances compensate income losses incurred by the receiving family during times of crises i.e., during recessions. If remittances are countercyclical, governments in developing countries can use remittances as part of a package of stabilization policies to manage the economy. In contrast, if remittances are pro-cyclical, there is an amplification effect of up-turns and down-turns. An extended nonlinear autoregressive distributed lag model is thus applied to estimate the asymmetric relationship between real GDP and remittances [8]. The cyclical components are extracted using the Hodrick-Prescott filter.

**Fig. 1:**
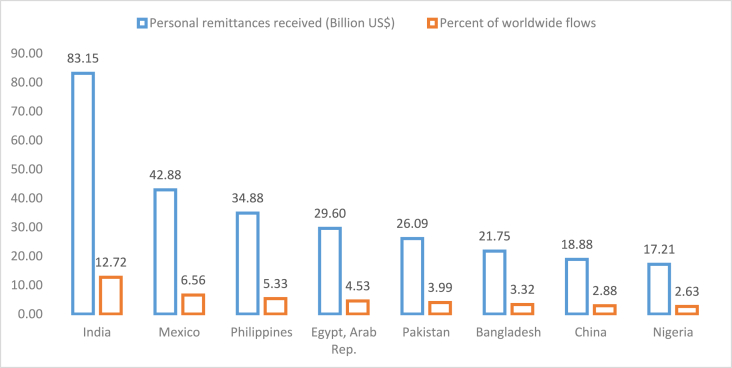
Remittance flows in the top eight receiving developing countries.

Source: World Development Indicators Database.

We consider an eclectic mix of eight developing countries which are amongst the top remittance-receiving countries in the world. Collectively, these countries account for about 42 percent of worldwide remittance flows in 2020 (Fig. 1). In all eight countries, we find that a decline in remittances will reduce output in expansions. Second, an increase in remittances will increase output during recessions. Positive remittance shocks are not linked with positive output shocks. Negative remittance shocks reduce output for Bangladesh, China, Mexico, and Nigeria in recessions only. The results suggest that remittances help the receiving country minimize the harmful effects of recessions confirming the insurance hypothesis. The results also indicate that remittances can prevent the receiving economy from overheating if it falls in expansions. Notably, these conclusions are consistent across the countries considered for analysis. In Mexico, the effect of remittances on economic activity is 4.8 times stronger in recessions when remittances rise compared to expansions when remittances fall. For the other countries, the effect of the decline in remittances on economic activity in expansions is 1.37 times stronger than the effect of an increase in remittances during recessions.

This study contributes to the literature on remittances and business cycles by stressing that the relationship between remittances and output is asymmetric as well as conditional on the face of the business cycle and the direction of change of remittances. For example, positive remittance shocks and positive output shocks are not related. However, negative remittance shocks reduce output in expansions. Similarly, positive remittance shocks increase output in a recession. Nevertheless, there is no discernible pattern in the relative strengths of the effect of negative remittance shocks in expansions, against positive remittance shocks in recessions. Comparable to Ref. [6], ignoring whether remittances are increasing or decreasing in expansions vs. contractions may yield biased results on the association between remittances and output. The findings generally differ from earlier research on the association between remittances and business cycles such as [4] by showing that while the association between remittances and output is indeed asymmetric, the relationship is contingent on the phase of the business cycle considered. The managerial implications are twofold and relate to the countercyclical nature of remittances. First, policymakers can use remittances to counteract recessions in the receiving country. Second, if remittances are maintained in expansions, future recessions can be delayed from occurring based on the results.

This study is set as follows. Section 2 reviews the literature on remittances and business cycles, and Sections 3, 4 discuss the methods and results, respectively. Section 5 concludes with policy implications.

## 2 Literature review

The counter-cyclicality of remittances can be drawn from the NELM framework [7]. According to the insurance hypothesis, remittances diversify the income sources of the receiving household to protect against negative income shocks during crisis periods [9]. The insurance hypothesis implies that remittances would increase with greater volatility of household income, the latter which is observed in recessions [10]. Thus, positive changes in remittances are more likely in crisis periods [11,12]. Because remittances increase the purchasing power of the receiving households [13], they spur aggregate demand in recessions. The insurance hypothesis implies that remittances are counter-cyclical capital flows [2,3,14] when remittances increase during recessions. By extension, remittances are expected to fall in expansions [11,12] inhibiting the growth of aggregate demand in the process.

How positive remittance shocks in expansions, and negative remittance shocks in recessions affect output is not explained by the insurance hypothesis. Insights can however be drawn from NELM's investment hypothesis. This hypothesis predicts that remittances are allocated towards productive income-generating assets [9]. Investments are highly pro-cyclical and abundant in expansions because their cash flows are known with certainty [15]. Thus, if the investment effect is dominant, positive remittance shocks would be expected in expansions, which would then trigger an increase in aggregate demand. By extension, if the investment motive prevails, remittances would fall during a recession further depressing aggregate demand in the process. Nonetheless, the empirical literature indicates that remittances have weak effects on productive allocations [9,16].

Research provides evidence against the counter-cyclicality of remittances. Pro-cyclicality is found [16] in India and [17] in Pakistan. On the other hand [18], find that remittances are a-cyclical by observing that income contraction in the home country does not induce the migrant to remit more in a sample of 122 countries. Similarly [19], find that remittances are a-cyclical in about 80 percent of their sample of 109 countries and that remittances appear to be pro-cyclical in the high remittance-receiving economies such as India and China. The a-cyclicality of remittances is confirmed in Nigeria using the ARDL model by Ref. [20]. Moreover [21], find that remittances do not dampen the negative effects of volatility in a panel of 142 countries. Both groups of studies provide evidence against the purported counter-cyclicality of remittances.

Counter-cyclicality of remittances is confirmed by Ref. [22] in the Philippines stating that remittances supplant about 60 percent of household income in monsoon seasons. This supports [2] who use data on border enforcement and macroeconomic indicators and develop a two-country DSGE business cycle model of labor migration. Their model matches the counter-cyclical dynamics and insurance role of remittances in consumption smoothing. Further evidence on counter-cyclicality is presented by Ref. [23] who find that remittances are countercyclical flows and promote risk-sharing (income smoothing) behavior in the less developed MENA countries but not in the oil-rich Gulf region. Extending this [24], confirm that remittances are countercyclical flows and promote risk-sharing behavior in a sample of 86 developing countries. Further support for the risk-sharing hypothesis is presented by Ref. [25] for a broad sample of countries that find that remittances are associated with increased risk-sharing behavior. Counter-cyclical remittances moderate the decline of household consumption in recessions [2]. This is because remittances are used to start up microenterprises that bolster household income during downturns [2].

The current empirical literature on remittance and cycles does not consider the role of asymmetric effects [26], although there is some evidence that the cyclicality of remittance can change in expansions and recessions [4,5,27]. According to Refs. [28,29], asymmetric effects in business cycles arise due to convex aggregate supply curves. During recessions, a shift in aggregate demand would have a stronger effect on output than prices because aggregate demand shifts on the flatter part of the supply curve [28,29]. However, the same shift in demand would have a stronger effect on prices than output in expansions because aggregate demand shifts on the vertical part of the supply curve. We can, therefore, infer that remittances will have asymmetric effects on output during expansions and recessions based on whether remittances increase or decrease [28,29]. To the best of our knowledge, no study has examined the asymmetric effects of positive and negative remittance shocks on output during expansions and recessions, respectively.

## 3 Theoretical framework

The New Economics of Labour Migration (NELM) serves as the theoretical foundation of this study [7,9]. The NELM is based on three underlying hypotheses. These are.

• Relative deprivation hypothesis: remittances go to those households that need it the most [7,9]. Empirical evidence by Ref. [9] however indicates that the relative deprivation hypothesis may not hold due to selection issues which suggest that only relatively wealthier households can afford to send family members overseas, and therefore, are more likely to receive remittances as compensation for the investment they made.
• Investment hypothesis: remittances are used by recipient households to engage in investments intended to generate income [7,9]. Empirical support is provided by Ref. [9].
• Insurance hypothesis: remittances are received in greater amounts in crisis periods to quell the effects of adverse situations such as natural disasters or economic recessions [9].

For this study, we follow [9] who notes that the relative deprivation hypothesis has limited empirical support. The insurance hypothesis suggests that remittances may be received more in crisis periods such as recessions. The investment hypothesis suggests that remittances are used for investment purposes. These two hypotheses pave the way for potential asymmetric effects of remittances in business cycle expansions and recessions. For example, in recessions, incomes are low, hence, the marginal impact of an additional dollar of remittance received may be stronger than in expansionary periods. This reasoning tells us that in recessions, a decline in remittances could have a stronger effect on recessions than an equivalently sized increase in remittances in recessionary periods. However, the caveat is that incomes may already be depressed in recessions and may, therefore, not fall much further due to a decline in remittances.

Business confidence and investment potential increase in economic expansions. The investment hypothesis may therefore dominate in expansionary periods. However, the additional investments may eventually be subject to diminishing returns consistent with neoclassical growth theory which predicts that the growth effects of investments eventually taper off. This suggests that any increase in remittances in expansions will have successively smaller effects on output.

## 4 Research design

### 4.1 Data

We extract the relevant data for analysis from the World Development Indicators database [30]. All the required data are measured in constant 2015 US dollars. Data for real GDP per-capita, broad money, and investments are available from 1960 to 2022 for all countries. Data for personal remittances received is available from 1982 to 2020 for China, 1978 to 2020 for India, 1979 to 2020 for Mexico, 1977 to 2020 for the Philippines, Egypt, and Nigeria, and 1976 to 2020 for Pakistan and Bangladesh. Data for foreign direct investments are available from 1979 to 2020 for China, 1975 to 2020 for India, 1970 to 2020 for Mexico, the Philippines, Egypt, Pakistan, and Nigeria, and from 1972 to 2020 for Bangladesh. Official aid data is available from 1979 to 2019 for China, 1978 to 2019 for India, 1981 to 2020 for Nigeria, and from 1960 to 2020 for the other countries. We use a sample of 1982–2019 for China, 1978 to 2019 for India, 1979 to 2020 for Mexico, 1977 to 2020 for the Philippines and Egypt, 1976 to 2020 for Pakistan and Bangladesh, and from 1981 to 2020 for Nigeria. All estimations are done in the Eviews 10 software package.

### 4.2 Model

Macroeconomic variables such as remittances experience repeated cyclical fluctuations around their long-run growth trends [6]. Business cycles refer to economy-wide fluctuations in production involving shifts between periods of rapid economic growth i.e., economic expansions, and periods of low economic growth or decline i.e., recessions. However, the accuracy of business cycles has been called into question given that fluctuations in economic activity do not follow a predictable pattern [31]. Macroeconomic data can thus be decomposed into the following unobserved trend and stationary cyclical components in equation (1):

(1) $$x=x^{\rho }+x^{T}$$

where $x^{\rho }$ is the permanent component of x, and $x^{T}$ is the stationary transitionary/cyclical component which represents fluctuations of x around the long-run permanent trend component.

The cyclical component is extracted using the Hodrick-Prescott filter. The HP filter assumes that a seasonally adjusted time series can be decomposed into permanent and cyclical components. To derive the decompositions, the HP filter minimizes the following loss function:

(2) $$x_{t}^{T}=x_{t}-\min\left\{\sum_{t=1}^{n}{\left(x_{t}-x_{t}^{\rho }\right)}^{2}+\lambda \sum_{t=2}^{n-1}{\left(\left(x_{t+1}^{\rho }-x_{t}^{\rho }\right)-\left(x_{t}^{\rho }-x_{t-1}^{\rho }\right)\right)}^{2}\right\}$$

where the variables are previously defined, and λ is the smoothing parameter which is set to 100 for annual data. The λ coefficient penalizes the variation of the long-run trend term from one period to the next. If λ=0, the time-varying trend is not discriminated against the actual series, and the cyclical components are minimized, whereas if λ=∞*,* the trend term is linear in equation (2). This would indicate that the permanent component follows a linear trend and is not time-varying.

To model the cyclical association between remittances and output, we estimate equation (3) below:

(3) $$\ln\;y_{t}^{T}=\pi +\beta^{T}\;\ln\;{rm}_{t}^{T}+control\;variables+u_{t}$$

where $y_{t}^{T}$ is the cyclical GDP per-capita, ${rm}_{t}^{T}$ is the cyclical component of remittances, and $u_{t}$ is the error term. The coefficient $\beta^{T}$ measures the effect of the transitionary component of remittances on the transitionary component of real GDP per capita. A positive value indicates that transitionary growth in remittances increases real GDP per capita above its trend value fueling an expansion. A negative value indicates that positive transitionary changes in remittances reduce output below its trend value fueling a recession.

We include the following control variables in our analysis. First, we identify and control for structural breaks using the Bai-Perron break test [[32], [33], [34]]. The benefits of the approach include its ability to detect multiple breaks. The break dates can be correlated with actual events to provide insights into the cause of the breaks. Structural breaks are measured using dummy variables set to 1 from the identified break date.

### 4.3 Control variables

We follow [35] and include the following control variables in our analysis: financial development (fd), investments (in), foreign direct investments (fdi), and aid (aid). We measure financial development using the log of liquid liabilities. These control variables are justified by Refs. [25,27,35]. For example, financial development and investments are underscored as channels through which remittances may impact economic activity [25,27,35]. Foreign direct investments and aid are alternative capital flows that could act as substitutes for the economic effects of inward remittances [25,27,35]. These variables may therefore determine the effect of remittances on economic activity [25,27,35].

### 4.4 MNARDL models

To estimate the transitionary models, we use a general to specific procedure to determine the appropriate lags to model the effect of explanatory variables on transitionary output [36]. We follow [8] and extend the approach of [36] to allow for the examination of asymmetric effects across business cycles. We thus use the extended nonlinear autoregressive distributed lag (MNARDL) approach to model the effects of positive and negative remittance shocks on positive and negative output shocks [8]. This allows us to examine the joint cases of asymmetric effects arising from the state of the economy, whether in recession or expansion, and the direction of change of remittances. Because we are dealing with stationary transitionary data, we estimate the extended NARDL model in levels like [36]. The extended NARDL model allows us to introduce a lag structure in the cyclical effect of remittances on GDP. The NARDL model allows us to estimate the effect of test variables on the outcome variable without pretesting for unit roots, which is particularly useful in our case as the cyclical elements are likely to be stationary [8]. Moreover, the NARDL model has the added benefit of avoiding the endogeneity bias as long as the estimated model is statistically free from the effects of autocorrelation [8]. Second, the lagged structure mitigates endogeneity concerns provided the estimated residuals are free from serial correlation.

Following [8], we delineate positive and negative changes in the data using the partial sum decomposition approach in equations (4), (4.1), (4.2) as follows. Thus, we have:

(4) $$x_{t}^{T}=x_{0}^{T}+x_{t}^{T^{+}}+x_{t}^{T^{-}}$$

where:

(4.1) $$x_{t}^{T^{+}}=\sum_{j=1}^{t}\Delta x_{t}^{T^{+}}=\sum_{j=1}^{t}\;\max\;\left({{\Delta x}_{j}}^{T},0\right)$$

and

(4.2) $$x_{t}^{T^{-}}=\sum_{j=1}^{t}\Delta x_{t}^{T^{-}}=\sum_{j=1}^{t}\;\min\;\left({{\Delta x}_{j}}^{T},0\right)$$

where $x_{t}^{T}$ is the transitionary component of $x_{t}$, $x_{0}^{T}$ is an arbitrary value, $x_{t}^{T^{+}}$ and $x_{t}^{T^{-}}$ are the partial sum processes which accumulate positive and negative changes in $x_{t}^{T}$.

The expansionary models are presented in equations (5.1), (5.2) below:

(5.1) $$\ln\;y_{t}^{T^{+}}=\alpha_{1}+\sum_{j=1}^{k}\alpha_{2j}\;\ln\;y_{t-j}^{T^{+}}+\sum_{j=0}^{k}\alpha_{3j}\;\ln\;{rm}_{t-j}^{T^{+}}+u_{1,t}$$

and

(5.2) $$\ln\;y_{t}^{T^{+}}=\mu_{1}+\sum_{j=1}^{k}\mu_{2j}\;\ln\;y_{t-j}^{T^{+}}+\sum_{j=0}^{k}\mu_{3j}\;\ln\;{rm}_{t-j}^{T^{-}}+u_{2,t}$$

If $\alpha_{3j}\forall j$ is positive, positive changes in remittances increase positive changes in output. If $\mu_{3j}\forall j$ is negative, negative changes in remittances decrease positive changes in output.

The recessionary models are presented in equations (6.1), (6.2) below:

(6.1) $$\ln\;y_{t}^{T^{-}}=\beta_{1}+\sum_{j=1}^{k}\beta_{2j}\;\ln\;y_{t-j}^{T^{-}}+\sum_{j=0}^{k}\beta_{3j}\;\ln\;{rm}_{t-j}^{T^{-}}+\varepsilon_{1,t}$$

and

(6.2) $$\ln\;y_{t}^{T^{-}}=\eta_{1}+\sum_{j=1}^{k}\eta_{2j}\;\ln\;y_{t-j}^{T^{-}}+\sum_{j=0}^{k}\eta_{3j}\;\ln\;{rm}_{t-j}^{T^{+}}+\varepsilon_{2,t}$$

If $\beta_{3j}\forall j$ is positive, negative changes in remittances increase negative changes in output. If $\eta_{3j}\forall j$ is negative, positive changes in remittances decrease the negative changes in output.

## 5 Results and discussion

### 5.1 Nonlinearity test

To ascertain the appropriateness of the nonlinear decompositions, we first perform the BDS test of nonlinearity to determine whether the underlying time-series variables are independently and identically distributed. Rejection of this null hypothesis suggests plausible nonlinearity of the time series variables. Our results suggest that the variables may be subject to nonlinearity (Table 1).

**Table 1:** BDS nonlinearity test.

Variables	Bangladesh	China	Egypt	India	Mexico	Nigeria	Pakistan	Philippines
lny	0.1818^A^	0.2002^A^	0.2001^A^	0.1859^A^	0.1587^A^	0.1655^A^	0.1950^A^	0.1669^A^
lnrm	0.1889^A^	0.1233^A^	0.1325^A^	0.1795^A^	0.1622^A^	0.1819^A^	0.1511^A^	0.1967^A^
lnfd	0.1908^A^	0.2061^A^	0.1931^A^	0.1966^A^	0.1313^A^	0.1782^A^	0.1889^A^	0.1913^A^
lnfdi	0.1418^A^	0.2026^A^	0.1172^A^	0.1748^A^	0.1261^A^	0.1094^A^	0.1619^A^	0.0996^A^
lnaid	0.0533^A^	0.1183^A^	0.0869^A^	0.0551^C^	0.0828^A^	0.1694^A^	0.0293^A^	0.0672^A^
lnin	0.1967^A^	0.1994^A^	0.1892^A^	0.1881^A^	0.1646^A^	0.1545^A^	0.2011^A^	0.1669^A^

Note: A, C indicate significance at 1 % and 10 %. Results reported for 2 dimensions. Identical conclusions were reached for up to 6 dimensions.

### 5.2 Structural breaks

The results of the break test are presented in Table 2. A description of the underlying events of the breaks for the respective countries is sourced from BBC News country profiles [37].

**Table 2:** Break test.

Model	0 vs. 1 break	1 vs. 2 breaks	2 vs. 3 breaks	3 vs. 4 breaks	Break dates
Critical value	8.58	10.13	11.14	11.83	
***Panel a. Expansionary models with positive changes in remittances***(+,+)					
Bangladesh	11.7476	23.583	11.781	1.326	1990, 1996, 2007
Egypt	35.621	6.777			1995
Mexico	56.758	43.635	6.293		1986, 2011
Pakistan	18.579	6.177			1988
Philippines	12.511	11.276	2.776		2001, 2009
Nigeria	21.117	12.267	4.586		1990, 2002
***Panel b. Expansionary models with negative changes in remittances***(−,+)					
China	15.338	11.9357	13.799	0.457	1993, 1998, 2007
Egypt	17.729	17.495	16.030	1.792	1984, 1999, 2007
India	23.600	10.042			1991
Mexico	42.053	3.934			1996
Pakistan	27.320	19.664	3.857		1988, 2005
Philippines	74.552	22.494	3.151		1988, 2008
Nigeria	48.207	18.895	10.689		1990, 1995
***Panel c. Recessionary models with positive changes in remittances***(+,−)					
Bangladesh	21.075	7.542			2001
China	105.031	8.931			1990
Egypt	20.479	8.208			2012
India	15.455	7.359			2001
Mexico	13.627	23.495	18.567	1.827	2003, 2009, 2015
Pakistan	21.075	7.542			2001
Philippines	32.525	4.783			1985
Nigeria	11.131	6.947			1989
***Panel d. Recessionary models with negative changes in remittances***(−,−)					
Bangladesh	12.424	7.237			1989
China	19.059	6.246			1994
Mexico	26.860	14.817	1.180		2002, 2009
Pakistan	18.042	10.452	7.036		1993, 2008
Philippines	61.218	15.447	16.997	2.849	1992, 1999, 2006
Nigeria	18.460	8.213			1994

Notes: Critical values based on [34].

For the expansionary models with positive changes in remittances (Table 2, Panel A), three breaks are identified for Bangladesh, and all have positive effects. These are periods that resolved major political crises and unrest. In Egypt, the 1995 break could represent the effects of the Gama'a al-Islamiyah Islamic Group terrorist attacks. In Mexico, 1986 has a significant negative effect which could reflect the negative effects of the Mexicana Airlines Boeing 727 crash [38]. The break, 1988 for Pakistan has a positive effect which reflects the election of Benazir Bhutto into Parliament. Breaks for the Philippines in 2001 and 2009 have negative effects which reflects the suspension of the impeachment against President Estrada, and the global financial crisis.

For the expansionary models with negative changes in remittances (Table 2, Panel B), three breaks are identified for China, but only 1993 is significant and therefore retained in the estimation. The 1993 break is positively associated with output. This could reflect the beginning of the construction of the three Gorges Dam in China. Three breaks are identified for Egypt, and these are 1984, 1999, and 2007. All three have positive effects. The break in 1991 had a negative effect in India. This has a negative effect on output during expansions which may reflect the assassination of Rajiv Gandhi. The breaks for Mexico, Philippines, and Nigeria are all positive indicating the beneficial effects of quelling civil unrest for Mexico in 1996, and the Philippines in 1988 and 2008, respectively.

Since the dependent variables in the recessionary models, a negative coefficient of the indicator variable representing the structural break implies a positive effect on output. With positive changes in remittances (Table 2, Panel C), the year 2001 is identified as a break for Bangladesh which has a negative coefficient. This could reflect the effect of the 2001 general elections in Bangladesh and the installment of the Khaleda Zia Nationalist Party coalition government. The break for China in 1990 could reflect the economic gains from the opening of stock markets in Shanghai and Shenzhen. The break for India in 2001 may reflect the positive economic effects of India's ability to launch satellites into deep space noting their strong space research sector. The break for the Philippines in 1985 could reflect the effect of the “people's power” and the overthrowing of President Marcos. The break for Egypt in 2012 could reflect the negative effects of religious terror violence. The breaks for Mexico could reflect the open warfare between Mexico's rival drug gangs.

For the recessionary model with negative changes in remittances (Table 2, Panel D), the breaks for Bangladesh and Egypt are insignificant and excluded from estimation. The year, 1994, reflects the abolishment of the Chinese exchange rate from the floating rate to a lower fixed rate which would have not augured well for China's exports. The break for Nigeria in 1994 could reflect the effects of Nigerian Politician, Moshood Abiola proclaiming himself president in a failed coup attempt. The break for Mexico in 2002 could reflect the negative effect of the release of confidential secret security files shedding light on the repression of political activists in the 1960s and 1970s. The break in 2009 could reflect the effects of the ongoing war between the rival Mexican drug gangs. The break for Pakistan in 1993 could reflect the effects of the general elections in Pakistan. The break in 2008 could reflect the effects of the suicide bombing on the Marriot Hotel in Islamabad.

### 5.3 Main estimates

**Fig. 2:**
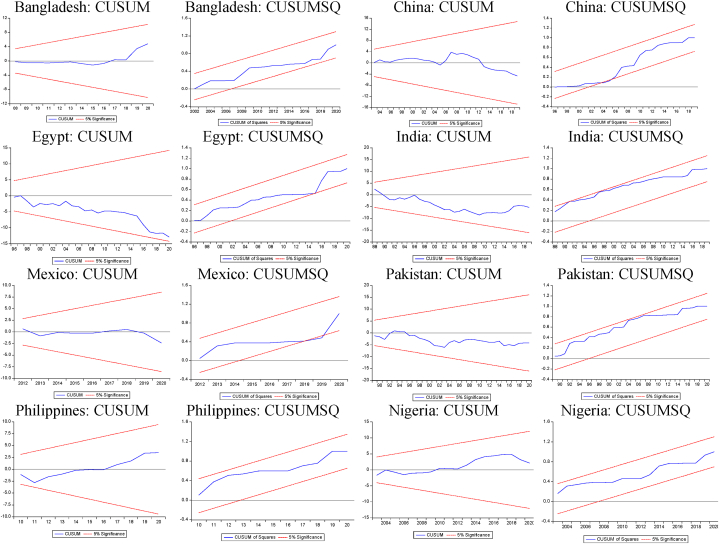
CUSUM and CUSUM SQ Plots (+,+). Note: CUSUM/CUSUMSQ plots within 5 % boundaries indicate stable estimates.

**Fig. 3:**
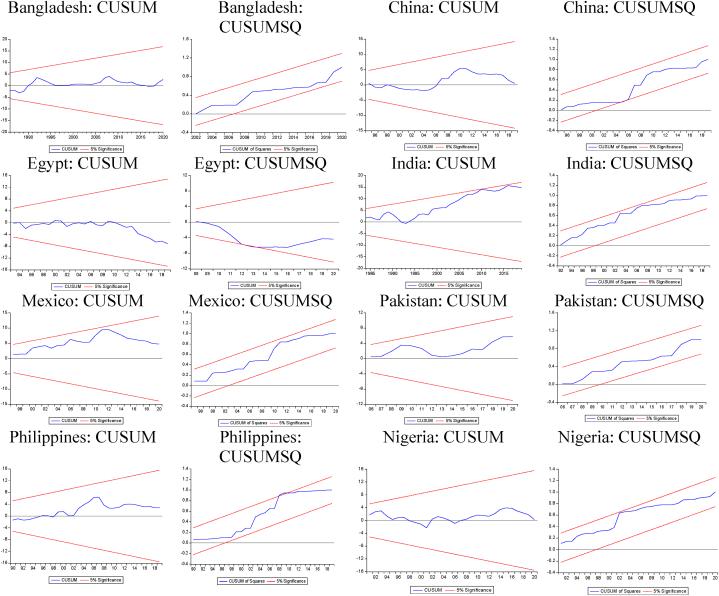
CUSUM and CUSUM SQ Plots (−,+). Note: CUSUM/CUSUMSQ plots within 5 % boundaries indicate stable estimates.

**Fig. 4:**
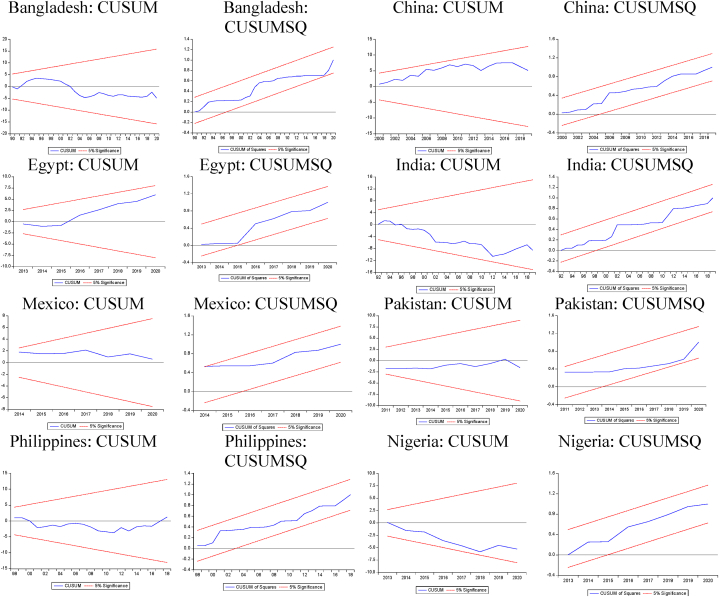
CUSUM and CUSUM SQ Plots (+,−). Note: CUSUM/CUSUMSQ plots within 5 % boundaries indicate stable estimates.

**Fig. 5:**
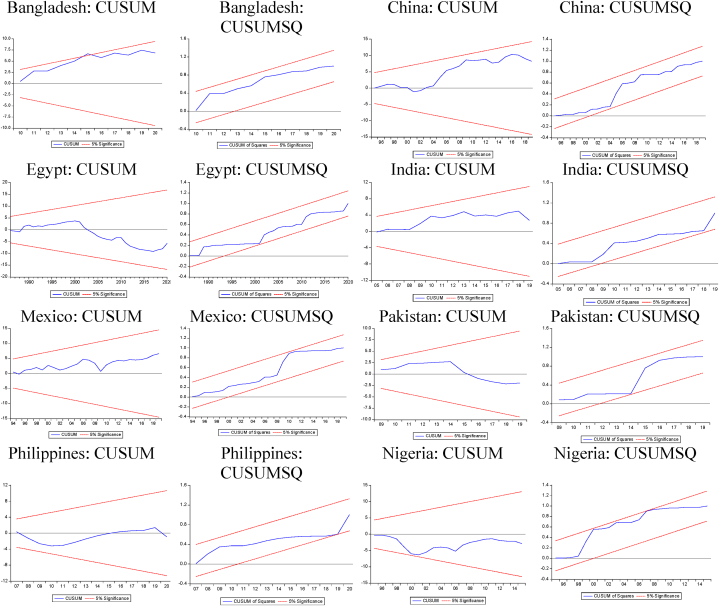
CUSUM and CUSUM SQ Plots (−,−). Note: CUSUM/CUSUMSQ plots within 5 % boundaries indicate stable estimates.

The extended NARDL estimates are presented in Table 3, Table 4, Table 5, Table 6 below. The data is stationary in levels using the nonlinear Flexible Fourier unit root test. The maximum lag length and optimum lag structure are determined by the Akaike information criteria. The estimated models satisfy the various diagnostic criteria on the absence of serial correlation and heteroscedasticity, presence of residual normality, correct functional form, and parameter stability. These results are based on the Breusch–Godfrey serial correlation test, Breusch–Pagan–Godfrey heteroscedasticity test, Jarque-Bera normality test, and the CUSUM and CUSUM of Squares stability test plots (Fig. 2, Fig. 3, Fig. 4, Fig. 5).

**Table 3:** Expansionary models with positive changes in remittances (+,+).

Variables	Bangladesh	China	Egypt	India	Mexico	Pakistan	Philippines	Nigeria
**Panel a. MNARDL estimates**								
$\ln\;y_{t-1}^{T+}$	0.0519 [0.0841]	0.6456^A^ [0.1074]	0.3668^A^ [0.0664]	0.3523^A^ [0.1072]	0.7173^A^ [0.1749]	0.3876^A^ [0.1127]	0.3356^A^ [0.1006]	0.5072^A^ [0.1316]
$\ln\;{rm}_{t}^{T+}$	0.0183^B^ [0.0091]	−0.0062 [0.0041]	−0.0040 [0.0031]	−0.0109 [0.0126]	0.0042 [0.0115]	−0.0084 [0.0067]	0.0240 [0.0150]	−0.0119^B^ [0.0034]
$\ln\;{rm}_{t-1}^{T+}$		0.0059 [0.0040]		0.0148 [0.0100]		0.0142^B^ [0.0069]		
$\ln\;{fd}_{t}^{T+}$	0.0073 [0.0078]	−0.0267 [0.0445]	−0.0031 [0.0112]	0.2383^B^ [0.0873]	0.0392^A^ [0.0139]	0.0086 [0.0236]	−0.0288 [0.0263]	0.0250 [0.0259]
$\ln\;{fd}_{t-1}^{T+}$					−0.0388^A^ [0.0130]			
$\ln\;{fdi}_{t}^{T+}$	−0.0001 [0.0009]	0.0259^A^ [0.0073]	−0.043^B^ [0.0026]	−0.002 [0.0017]	−0.0004 [0.0009]	0.0014 [0.0036]	−0.0007 [0.0014]	0.0143^B^ [0.0041]
$\ln\;{fdi}_{t-1}^{T+}$			0.0075^A^ [0.0024]					
$\ln\;{aid}_{t}^{T+}$	−0.0039 [0.0029]	−0.0033^C^ [0.0019]	−0.0017^B^ [0.0008]	0.0020 [0.0029]	0.0029 [0.0049]	0.0013 [0.0027]	0.0034 [0.0021]	0.0032 [0.0054]
$\ln\;{in}_{t}^{T+}$	0.2045 [0.0263]	0.1142^B^ [0.0416]	0.1087^A^ [0.0160]	0.0798^A^ [0.0227]	0.1929^A^ [0.0237]	0.1624^A^ [0.0273]	0.1097^A^ [0.0177]	0.0548 [0.0362]
$\ln\;{in}_{t-1}^{T+}$		−0.0722 [0.0428]			−0.1345^A^ [0.0452]	−0.0652^B^ [0.0305]		−0.0625 [0.0116]
Break 1	0.0130^A^ [0.0034]		−0.0147^A^ [0.0035]		−0.0182^B^ [0.0088]	0.0102^A^ [0.0047]	−0.0169^A^ [0.0061]	0.0661^A^ [0.0116]
Break 2	0.0063 [0.0063]				−0.0161 [0.0111]		−0.0371^A^ [0.0102]	0.1081^A^ [0.0179]
Break 3	0.0203^A^ [0.0071]							
Intercept	−0.0319^A^ [0.0091]	0.0172^A^ [0.0056]	−0.0089^B^ [0.0041]	0.0279^A^ [0.0051]	−0.0046 [0.0128]	0.0102^B^ [0.0047]	0.0316^A^ [0.0038]	0.0128^B^ [0.0064]
***Panel b. Diagnostic tests***								
Serial correlation	1.6365 (0.4412)	1.7963 (0.5376)	1.9653 (0.4967)	4.8359 (0.0891)	2.6257 (0.2690)	2.9382 (0.2301)	4.5805 (0.1012)	0.5668 (0.7532)
Heteroscedasticity	5.5265 (0.7862)	6.9634 (0.5964)	10.9415 (0.2051)	12.3221 (0.0905)	15.9437 (0.1013)	14.3620 (0.1100)	7.6819 (0.4651)	7.2779 (0.6082)
Normality	3.4223 (0.1806)	3.9862 (0.1909)	5.3769 (0.0679)	2.4003 (0.3011)	4.5172 (0.1201)	5.7258 (0.0571)	2.9655 (0.2270)	1.2859 (0.5257)
Functional form	1.8693 (0.1811)	0.0614 (0.8061)	1.0672 (0.2942)	0.9497 (0.3373)	1.0977 (0.3013)	0.0841 (0.7737)	0.9073 (0.3480)	1.2537 (0.2727)

Notes: A, B, C indicate significance at the 1, 5, and 10 percent levels, respectively. The null hypothesis for the diagnostic tests: absence of heteroscedasticity and serial correlation, normality of residuals, and correct function form. The respective p-values for the diagnostic tests are in (.). Two degrees of freedom are used for the serial correlation test. The normality and functional form tests each have one degrees of freedom. The degrees of freedom for the heteroscedasticity test depends on the number of regressors in the MNARDL model. The test-statistic reported for the diagnostic tests is the chi-squared test statistic.

**Table 4:** Expansionary models with negative changes in remittances (−,+).

Variables	Bangladesh	China	Egypt	India	Mexico	Pakistan	Philippines	Nigeria
**Panel a. MNARDL estimates**								
$\ln\;y_{t-1}^{T+}$	0.6088^A^ [0.1141]	0.7765^A^ [0.0975]	0.3665^A^ [0.1162]	0.6658^A^ [0.1189]	0.3889^A^ [0.1096]	0.5099^A^ [0.1147]	0.5435^A^ [0.0946]	0.1778 [0.1509]
$\ln\;y_{t-2}^{T+}$				−0.4086^A^ [0.1151]				
$\ln\;{rm}_{t}^{T-}$	0.0001 [0.0125]	−0.0064^C^ [0.0035]	−0.0077 [0.0060]	−0.0530^A^ [0.0123]	−0.0389^C^ [0.0197]	−0.0411^A^ [0.0108]	−0.0339^B^ [0.0146]	−0.0231^A^ [0.0064]
$\ln\;{rm}_{t-1}^{T-}$				−0.0207 [0.0130]				
$\ln\;{fd}_{t}^{T-}$	0.0093 [0.0264]	−0.0246 [0.0533]	0.0256 [0.0396]	0.0531 [0.0581]	0.0178 [0.0117]	0.0696^B^ [0.0267]	0.0029 [0.0435]	−0.0754^C^ [0.0435]
$\ln\;{fd}_{t-1}^{T-}$		−0.1114^C^ [0.0614]			−0.0153 [0.0111]			
$\ln\;{fdi}_{t}^{T-}$	−0.0004 [0.0013]	0.0567^A^ [0.0073]	−0.0121^A^ [0.0032]	−0.0109^A^ [0.0022]	−0.0104 [0.0087]	−0.0013 [0.0040]	−0.0006 [0.0026]	0.0057 [0.0078]
$\ln\;{fdi}_{t-1}^{T-}$				0.0102^A^ [0.0021]	−0.0138 [0.0089]			
$\ln\;{aid}_{t}^{T-}$	−0.0110^A^ [0.0038]	−0.0072^C^ [0.0032]	−0.0005 [0.0018]	−0.0089^C^ [0.0044]	−0.0043 [0.0041]	−0.0077 [0.0046]	−0.0127^A^ [0.0029]	0.0094 [0.0066]
$\ln\;{aid}_{t-1}^{T-}$			−0.0055^B^ [0.0022]					
$\ln\;{in}_{t}^{T-}$	0.0906 [0.0567]	0.0965^C^ [0.0485]	0.0461^A^ [0.0154]	−0.0300^C^ [0.0165]	0.0320^A^ [0.0112]	0.0375 [0.0293]	0.0095 [0.0132]	−0.0469 [0.0297]
$\ln\;{in}_{t-1}^{T-}$	−0.1112^C^ [0.0624]	−0.1602^A^ [0.0426]				−0.0159^C^ [0.0084]		
Break 1		0.0198^B^ [0.0075]	0.0242^A^ [0.0061]	−0.0251^A^ [0.0061]	0.0467^A^ [0.0075]	−0.0159^C^ [0.0084]	0.0400^A^ [0.0085]	0.0821^A^ [0.0181]
Break 2			0.0383^A^ [0.0094]				0.0201^C^ [0.0104]	
Break 3			0.0647^A^ [0.0137]					
Intercept	0.0249^A^ [0.0044]	0.0295^A^ [0.0049]	0.0214^A^ [0.0043]	0.0011 [0.0054]	0.0778^A^ [0.0092]	0.0111^A^ [0.0032]	0.0474^A^ [0.0094]	−0.0089 [0.0172]
***Panel b. Diagnostic tests***								
Serial correlation	0.4863 (0.7842)	0.1766 (0.9154)	1.4742 (0.4785)	2.9861 (0.0840)	0.1797 (0.9141)	0.7185 (0.7701)	5.1448 (0.0764)	0.4998 (0.7789)
Heteroscedasticity	10.1165 (0.1821)	15.1159 (0.0878)	6.6083 (0.5794)	9.1971 (0.5135)	4.6182 (0.8662)	9.8629 (0.3617)	10.4327 (0.2360)	11.5891 (0.1149)
Normality	2.7455 (0.2117)	0.3058 (0.8582)	3.6427 (0.1618)	0.6974 (0.7056)	0.7083 (0.7017)	3.5387 (0.1796)	0.1020 (0.9503)	0.2522 (0.8815)
Functional form	0.7830 (0.3824)	2.9770 (0.0968)	3.8907 (0.0572)	0.0912 (0.7650)	3.7608 (0.0589)	2.7419 (0.1075)	0.0001 (0.9896)	1.1800 (0.2863)

Notes: A, B, C indicate significance at the 1, 5, and 10 percent levels, respectively. The null hypothesis for the diagnostic tests: absence of heteroscedasticity and serial correlation, normality of residuals, and correct function form. The respective p-values for the diagnostic tests are in (.). Two degrees of freedom are used for the serial correlation test. The normality and functional form tests each have one degrees of freedom. The degrees of freedom for the heteroscedasticity test depend on the number of regressors in the MNARDL model. The test statistic reported for the diagnostic tests is the chi-squared test statistic.

**Table 5:** Recessionary models with positive changes in remittances (+,−).

Variables	Bangladesh	China	Egypt	India	Mexico	Pakistan	Philippines	Nigeria
**Panel a. MNARDL estimates**								
$\ln\;y_{t-1}^{T-}$	0.4065^A^ [0.1244]	0.4029^B^ [0.1775]	0.6451^A^ [0.0972]	0.3549^B^ [0.1446]	0.2269 [0.1341]	0.4223^A^ [0.1329]	0.1929^A^ [0.2148]	0.4052^A^ [0.0995]
$\ln\;{rm}_{t}^{T+}$	−0.0531^B^ [0.0116]	0.0031 [0.0064]	−0.0136^B^ [0.0063]	−0.0484^A^ [0.0121]	−0.2355^A^ [0.0358]	−0.0182^B^ [0.0077]	−0.0803^B^ [0.0391]	−0.0113^A^ [0.0034]
$\ln\;{rm}_{t-1}^{T+}$		−0.0123^C^ [0.0064]						
$\ln\;{fd}_{t}^{T+}$	0.0057 [0.0137]	−0.0682 [0.0633]	0.0434 [0.0376]	−0.0837 [0.1449]	−0.0187 [0.0180]	0.0167 [0.0321]	−0.0466 [0.0780]	0.0051 [0.0293]
$\ln\;{fd}_{t-1}^{T+}$				−0.3725^B^ [0.1368]				
$\ln\;{fdi}_{t}^{T+}$	−0.0001 [0.0008]	0.0186 [0.0116]	−0.0118^B^ [0.0029]	−0.0036 [0.0037]	−0.0098 [0.0127]	0.0084 [0.0054]	0.0022 [0.0056]	−0.0183^B^ [0.0074]
$\ln\;{fdi}_{t-1}^{T+}$				0.0067 [0.0043]				
$\ln\;{aid}_{t}^{T+}$	0.0013 [0.0036]	−0.0102^C^ [0.0039]	0.0003 [0.0019]	−0.0135^C^ [0.0072]	0.0027 [0.0092]	−0.0143^B^ [0.0065]	−0.0158 [0.0046]	0.0138^C^ [0.0068]
$\ln\;{aid}_{t-1}^{T+}$			−0.0045^C^ [0.0025]	0.0115 [0.0075]		−0.0093 [0.0057]		
$\ln\;{in}_{t}^{T+}$	0.0965^B^ [0.0444]	0.0197 [0.0604]	0.0545^A^ [0.0160]	0.0602^B^ [0.0283]	0.0827^C^ [0.0474]	0.0937^A^ [0.0386]	0.0269^A^ [0.0177]	0.0157 [0.0388]
$\ln\;{in}_{t-1}^{T+}$	−0.0480 [0.0331]					−0.1397^A^ [0.0373]		−0.0620 [0.0418]
Break 1	−0.0072^C^ [0.0042]	−0.0615^A^ [0.0157]	0.0120^B^ [0.0050]	−0.0141^C^ [0.0072]	0.0759^A^ [0.0126]	0.0211^B^ [0.0077]	−0.1082^A^ [0.0301]	0.0507^A^ [0.0154]
Break 2					0.0451^A^ [0.0129]			
Intercept	0.0363^A^ [0.0117]	−0.0143 [0.0092]	0.0179^A^ [0.0043]	−0.0441^A^ [0.0142]	0.2441 [0.0426]	−0.0202^B^ [0.0089]	0.0316^A^ [0.0038]	−0.1300^A^ [0.0170]
***Panel b. Diagnostic tests***								
Serial correlation	1.9972 (0.3684)	0.9492 (0.3299)	1.7211 (0.4229)	1.7187 (0.4234)	3.3773 (0.1848)	5.6145 (0.0604)	4.2609 (0.1188)	8.0949 (0.0882)
Heteroscedasticity	12.1525 (0.1445)	16.8316 (0.0514)	7.7785 (0.4554)	2.5351 (0.9904)	13.1335 (0.1196)	15.4478 (0.0793)	16.0178 (0.0673)	8.0906 (0.4247)
Normality	0.0915 (0.9552)	0.3059 (0.8581)	2.9508 (0.2286)	4.4278 (0.1091)	2.9668 (0.2246)	4.3145 (0.1156)	2.9798 (0.2310)	3.1712 (0.2048)
Functional form	0.8876 (0.3530)	1.4429 (0.2409)	0.4401 (0.5118)	0.1434 (0.7078)	0.1585 (0.6933)	3.0962 (0.0880)	1.1840 (0.2844)	3.0881 (0.0899)

Notes: A, B, C indicate significance at the 1, 5, and 10 percent levels, respectively. The null hypothesis for the diagnostic tests: absence of heteroscedasticity and serial correlation, normality of residuals, and correct function form. The respective p-values for the diagnostic tests are in (.). Two degrees of freedom are used for the serial correlation test. The normality and functional form tests each have one degrees of freedom. The degrees of freedom for the heteroscedasticity test depends on the number of regressors in the MNARDL model. The test-statistic reported for the diagnostic tests is the chi-squared test statistic.

**Table 6:** Recessionary models with negative changes in remittances (−,−).

Variables	Bangladesh	China	Egypt	India	Mexico	Pakistan	Philippines	Nigeria
**Panel a. MNARDL estimates**								
$\ln\;y_{t-1}^{T-}$	0.4819^A^ [0.1245]	0.2769^A^ [0.0764]	0.5794^A^ [0.1013]	0.1267 [0.1351]	0.4508^B^ [0.1713]	0.0633^A^ [0.1293]	0.0249 [0.0413]	0.2729^A^ [0.1420]
$\ln\;{rm}_{t}^{T-}$	−0.0191^C^ [0.0109]	−0.0041^B^ [0.0017]	−0.0111^C^ [0.0058]	−0.0075 [0.0139]	0.0060 [0.0214]	0.0205^A^ [0.0108]	0.0066 [0.0094]	−0.0111^C^ [0.0064]
$\ln\;{rm}_{t-1}^{T-}$					−0.0455^C^ [0.0233]			
$\ln\;{fd}_{t}^{T-}$	0.0373 [0.0229]	0.0211 [0.0319]	0.0629^C^ [0.0339]	−0.1515 [0.0779]	0.0122 [0.0080]	−0.0322 [0.0244]	−0.1203^A^ [0.0331]	0.0184 [0.0339]
$\ln\;{\text{fdi}}_{t}^{T-}$	−0.0011 [0.0019]	0.0179 [0.0126]	0.0022 [0.0026]	0.0036 [0.0019]	0.0028 [0.0057]	0.0034 [0.0036]	0.0017 [0.0016]	0.0194^B^ [0.0078]
$\ln\;{\text{aid}}_{t}^{T-}$	0.0059^C^ [0.0033]	−0.0059^C^ [0.0027]	−0.0027 [0.0016]	0.0135^B^ [0.0061]	−0.0009 [0.0043]	−0.0075^C^ [0.0046]	−0.0001 [0.0010]	−0.0067 [0.0065]
$\ln\;{\text{aid}}_{t-1}^{T-}$					0.0054 [0.0041]			
$\ln\;{\text{in}}_{t}^{T-}$	0.2600^A^ [0.0471]	0.2715^A^ [0.0400]	0.0583^A^ [0.0183]	0.1630^A^ [0.0232]	0.2146^A^ [0.0136]	0.1826^A^ [0.0219]	0.2157^A^ [0.0096]	0.0720^A^ [0.0394]
$\ln\;{\text{in}}_{t-1}^{T-}$	−0.1020 [0.0611]				−0.1011^C^ [0.0055]			
Break 1		0.0270^B^ [0.0056]		0.0045 [0.0061]	−0.0110^C^ [0.0056]	−0.0236^A^ [0.0049]	−0.0163^A^ [0.0027]	0.0821^A^ [0.0181]
Break 2					−0.0356^A^ [0.0119]	−0.0424^A^ [0.0085]	0.0345^A^ [0.0040]	
Intercept	−0.0138^A^ [0.0040]	0.0081^B^ [0.0035]	−0.0055 [0.0036]	−0.0709^A^ [0.0126]	0.0179^A^ [0.0057]	−0.0402^A^ [0.0047]	−0.001 [0.0069]	−0.1016^A^ [0.0188]
***Panel b. Diagnostic tests***								
Serial correlation	0.9270 (0.6291)	3.6892 (0.1581)	0.9185 (0.6317)	0.0496 (0.9775)	4.4480 (0.1082)	2.4621 (0.2920)	5.0918 (0.0576)	5.5162 (0.0634)
Heteroscedasticity	15.9237 (0.1112)	2.3796 (0.9925)	9.3488 (0.1549)	4.4905 (0.7219)	19.0810 (0.0597)	13.7661 (0.0881)	13.3353 (0.1008)	5.6728 (0.5784)
Normality	0.1915 (0.7652)	0.5665 (0.7533)	1.7065 (0.4260)	3.0827 (0.2140)	3.9926 (0.2096)	2.7300 (0.2553)	4.7025 (0.0952)	5.5445 (0.0597)
Functional form	1.3476 (0.2538)	2.6349 (0.1176)	1.5201 (0.2261)	1.0199 (0.3204)	1.5556 (0.2119)	3.6143 (0.0660)	1.2662 (0.2668)	3.7756 (0.0557)

Notes: A, B, C indicate significance at the 1, 5, and 10 percent levels, respectively. The null hypothesis for the diagnostic tests: absence of heteroscedasticity and serial correlation, normality of residuals, and correct function form. The respective p-values for the diagnostic tests are in (.). Two degrees of freedom are used for the serial correlation test. The normality and functional form tests each have one degrees of freedom. The degrees of freedom for the heteroscedasticity test depends on the number of regressors in the MNARDL model. The test-statistic reported for the diagnostic tests is the chi-squared test statistic.

Positive remittance shocks are not related to positive output shocks (Table 3) except in Bangladesh and the Philippines which show a small positive effect, and Nigeria where a negative effect is observed. Financial development, foreign direct investments, and aid are mostly insignificant. Negative remittance shocks are negatively associated with positive output shocks (Table 4). The association is insignificant for Bangladesh. Positive changes in remittances are negatively associated with negative changes in output in all eight countries (Table 5). Negative remittance shocks reduce output for Bangladesh, China, Mexico, and Nigeria, and were observed insignificant for the rest. The coefficient of negative changes in investments is positive and significant for all countries. Positive investment and output shocks (Table 5), and negative investment and output shocks (Table 6) are positively associated confirming the pro-cyclicality of investments [15]. Financial development, foreign direct investments, and official aid are generally insignificant.

The estimates reveal that remittances act as a buffer by smoothing economic fluctuations. The estimates confirm the counter-cyclical nature of remittances as capital flows irrespective of whether the receiving country is in an expansion or recession [2,3]. The implication is that remittances promote risk-sharing behavior in our sample of countries [[23], [24], [25]]. Risk-sharing behavior is important because excessive consumption fluctuations transmitted through output shocks can have adverse implications on the accumulation of human and physical capital [39]. Improved risk-sharing enhances economic efficiency by exploiting the gains associated with specialization and economies of scale [40]. Remittances reduce economic volatility and can be linked to long-run economic growth. The results indicate that remittances can be used to counteract severe fluctuations in economic activity. The findings agree with the prior belief on the insurance role remittances during crisis periods. Therefore, remittances can be considered as counter-cyclical fiscal policy. However, it is possible that government expenditure is not always counter-cyclical and can instead be pro-cyclical [6,[41], [42], [43]]. What complicates the use of remittances as a stabilization tool, however, is that, unlike fiscal policy, remittances cannot be directly controlled by policymakers. Instead, the decision to remit is a largely personal decision taken by migrants to help their family members back home.

### 5.4 Further tests

As further tests, we model the effect of the permanent component of remittances on the permanent component of output using the fully modified least squares (FMOLS) technique (Table 7). Remittances have a negative effect on permanent output in Bangladesh, Egypt, and Nigeria. The strongest positive effects are observed in India, followed by the Philippines, Mexico, China, and Pakistan. We follow [44] and consider threshold effects. Linear effects are appropriate for China and Nigeria. For the latter, the nonlinear specification also indicates that remittances reduce permanent output. A U-shaped effect is appropriate in Bangladesh, India, Mexico, and the Philippines. The result for Bangladesh is consistent with [44] which indicates that remittances would increase economic growth after a threshold level of remittances has been passed. An inverted U-shaped effect is appropriate for Egypt which means that remittances increase economic growth up to the threshold value of remittances, after which growth declines.

**Table 7:** Effect of permanent component of remittances on permanent component of output.

Variables	Bangladesh	China	Egypt	India	Mexico	Pakistan	Philippines	Nigeria
**Panel a: linear model**								
$\ln\;{\text{rm}}_{t}^{P}$	−0.0546^A^ [0.0047]	0.0326^A^ [0.0095]	−0.0916^A^ [0.0061]	0.2676^A^ [0.0522]	0.0395^A^ [0.0037]	0.0093^A^ [0.0013]	0.1308^A^ [0.0244]	−0.0395^A^ [0.0027]
Controls	Yes	Yes	Yes	Yes	Yes	Yes	Yes	Yes
***Panel b: nonlinear model***								
$\ln\;{\text{rm}}_{t}^{P}$	−0.7709^A^ [0.0672]	−0.2088 [0.1568]	0.8667^A^ [0.2548]	−5.2131^A^ [0.9840]	0.3454^A^ [0.0898]	−0.3500^A^ [0.1251]	−0.7476^A^ [0.2175]	−0.0588^A^ [0.0169]
$\ln\;{\text{rm}}_{t}^{P}\times \ln\;{\text{rm}}_{t}^{P}$	0.0177^A^ [0.0017]	0.0058 [0.0039]	−0.0204^A^ [0.0054]	0.1086^A^ [0.0196]	−0.0068^A^ [0.0019]	0.0081^A^ [0.0028]	0.0185^A^ [0.0046]	0.0004 [0.0004]
Controls	Yes	Yes	Yes	Yes	Yes	Yes	Yes	Yes
***Panel c: Engle-Granger cointegration test***								
Linear	−4.2136^A^	−2.3625^B^	−3.9602^A^	−2.8851^A^	−6.5001^A^	−4.2029^A^	−3.5840^A^	−3.3187^A^
Nonlinear	−5.3691^A^	−2.2208^B^	−4.2150^A^	−4.3383^A^	−3.4100^A^	−3.4907^A^	−3.0332^A^	−3.0169^A^

Notes: A, B, C indicate significance/cointegration at the 1, 5, and 10 percent levels, respectively. Standard error in [.]. The Engle-Granger test is conducted by running a unit root test on the estimated residuals of the cointegrating model estimated by FMOLS. The test assumes no deterministic components, and the optimal lag length is determined by the Akaike information criteria.

## 6 Conclusions, policy implications, and research outlook

In this study, we estimate the asymmetric effect of remittances on business cycles. We consider the top eight remittance-receiving developing countries based on the volume of remittances received in 2020. Collectively, these countries accounted for about 42 percent of remittance flows worldwide in 2020. Using nonlinear methods, we model the effects of remittances on output in expansions and recessions considering whether remittances are increasing or decreasing. We find that negative remittance shocks reduce output in expansions. Second, positive remittance shocks increase output in recessions. These findings are consistent for all eight countries. Positive remittance shocks are not related to output in expansions. Similarly, negative remittance shocks are not linked with output in recessions. The combined results indicate that remittances are counter-cyclical capital flows when remittances decrease in expansions, and when they increase in recessions.

The practical policy implication is that remittances can be used to smoothen income fluctuations and promote risk-sharing behavior in the top eight remittance-receiving countries. Although positive changes in remittances do not increase output in expansions, remittances can help policymakers prolong the expansionary phase of the business cycle provided remittances do not decline in expansions. Therefore, in expansions, policymakers should devise economic policies in sync with money transfer operators to avoid any decline in remittances. In recessions, the appropriate policy recommendation is to reduce money transfer fees to encourage a greater volume of remittances. Based purely on the results, remittances can be used as part of a package of stabilization policies such as counter-cyclical fiscal policies assuming that recessions and expansions in the sender and receiver countries are not perfectly synchronized [6].

As our results support counter-cyclicality, one implication from our research is that remittances promote risk-sharing behaviour in our sample countries. However, we do not quantify the extent of risk-sharing behaviour from remittances. Future research can, therefore, explore this dimension in the top eight remittance-receiving countries. Second, based on the insurance hypothesis, our research rests on the assertion that remittances are more likely to decline in expansions and increase in recessions. We provide support for this assumption because negative changes in remittances significantly reduce output in expansions. Similarly, positive changes in remittances significantly increase output in recessions. Future research can explore the validity of this claim using survey-level data on when migrants are more likely to send more/less remittances.

Nevertheless, the limiting factor of our research is that only eight countries are employed as case studies. Although these countries are amongst the largest recipients of remittances, as the data and analysis indicate, the economic activities in countries differ and therefore, any generalization of our results to other countries must be treated with caution. It is possible that the hypothesized relationship may differ in other groups of countries or may differ in the region. Another limitation is that structural breaks are identified using the Bai-Perron approach, which although can be applied with cyclical or stationary data, may only provide indications of up to 5 structural breaks. However, this is an arbitrary limit. A more updated methodology to model the effects of structural breaks is the Fourier autoregressive distributed lag approach developed by Banerjee et al. [45] which can accommodate the effects of numerous structural breaks using the Fourier function. The other limitation is that the cyclical components are extracted using only the Hodrick-Prescott filter. Future research can consider this updated approach as well as many other countries as test samples to generalize the conclusions derived from our study. Future research can build on this framework and explore alternative techniques to extract business cycles, and potentially, model the cyclical determinants of remittances in developing countries.

## CRediT authorship contribution statement

**Nikeel Nishkar Kumar:** Writing – original draft, Software, Methodology, Formal analysis, Conceptualization. **Rajesh Mohnot:** Writing – review & editing, Validation, Supervision, Project administration, Methodology, Investigation, Conceptualization.

## Data availability statement

The data used for analysis will be made available upon request.

## Declaration of competing interest

The authors declare that they have no known competing financial interests or personal relationships that could have appeared to influence the work reported in this paper.
